# Toward precision neuromodulation in ADHD: rTMS-evoked excitability as a biomarker and ccPAS as an alternative protocol

**DOI:** 10.1016/j.nicl.2026.104067

**Published:** 2026-09-24

**Authors:** Uri Alyagon, Lihi Bokovza, Hadar Shalev, Noam Ygael, Abraham Zangen

**Affiliations:** a Department of Life Sciences and the Zelman Center for Brain Science Research, Ben-Gurion University of the Negev, Beer-Sheva, Israel; b Psychiatry Department, Soroka Medical Center, Beer-Sheva, Israel

**Keywords:** Repetitive transcranial magnetic stimulation, Attention deficit hyperactivity disorder, rTMS evoked biomarker, Cortico-cortical paired associative stimulation

## Abstract

Attention Deficit Hyperactivity Disorder (ADHD) is associated with cognitive and emotional-motivational dysregulation and with right prefrontal cortex (rPFC) dysfunction. While repetitive transcranial magnetic stimulation (rTMS) has shown promise in alleviating ADHD symptoms, treatment outcomes are not consistent or robust. This study was designed to initiate the prospective validation process of a recently suggested electrophysiological biomarker for clinical response to high-frequency rTMS (HF-rTMS) in adults with ADHD. For biomarker non-compatible patients, the study explored the safety and efficacy of a novel treatment protocol using cortico-cortical Paired Associative Stimulation (ccPAS) as an alternative stimulation approach. Fifty-eight TMS-naive adults diagnosed with ADHD underwent biomarker screening to characterize the clinical profile of biomarker-compatible and non-compatible participants. Thereafter, a subgroup of biomarker-compatible participants (*n* = 15) underwent treatments with HF-rTMS (15 sessions over 3 weeks), while non-compatible participants were randomized to one of two ccPAS protocols with different order of hemispheric stimulation (left-to-right: *n* = 12; right-to-left: *n* = 12). Biomarker-compatible participants exhibited significantly lower baseline ADHD symptom severity (d_Cohen_ = 0.46) and elevated response inhibition (d_Cohen_ = 0.53) relative to non-compatible participants. Furthermore, symptom severity in the compatible subgroup was associated with alpha-band resting-state asymmetry (*r* = 0.65). Notably, compared to an existing dataset of participants who were randomly allocated to active (*n* = 15) and sham (*n* = 14) treatment, biomarker-allocated HF-rTMS participants exhibited enhanced clinical outcome, both immediately after the treatment and at a one-month follow-up (η^2^_partial_ = 0.08–0.12). Conversely, ccPAS treatment yielded modest symptom reduction in the two groups with no significant effect of stimulation order (η^2^_partial_ = 0.08), suggesting that treatment outcome was not influenced by the associative mechanism of the ccPAS. These findings provide preliminary support for the potential utility of rTMS-evoked excitability as a biomarker for personalized ADHD treatment and underscore the need for further investigation into ccPAS as a therapeutic alternative.

## Highlights

• rTMS-evoked biomarker was associated with lower ADHD severity and enhanced response inhibition.
• Biomarker-allocated HF-rTMS participants had enhanced outcomes versus random allocation.
• ccPAS was safe but did not improve outcome in biomarker non-compatible participants.

## 1 Introduction

Affecting 7.6% of children, 5.6% of teenagers (Salari et al., 2023) and 3.1% of adults worldwide (Ayano et al., 2023), Attention Deficit Hyperactivity Disorder (ADHD), characterized by poor attention, impulsivity, hyperactivity and emotional-motivational dysregulation (Sonuga-Barke, 2005), introduces a substantial social and financial burden (Sciberras et al., 2022). Although pharmacological remedies are available, 50% of patients do not find them beneficial or discontinue use mainly due to diverse side effects (Gajria et al., 2014), warranting the development of alternative treatments and methods to promote response rate. One such alternative is transcranial magnetic stimulation (TMS), which may induce long-term alleviation of symptoms by targeting the underlying neuropathology.

ADHD is associated with deficits in multiple domains of executive functions (Willcutt et al., 2005), and in accordance, with functional and structural abnormalities, mainly of frontal networks (Rubia et al., 2014), and specifically right sided ones. For example, brain morphometry and fMRI studies during attentional tasks found that the right prefrontal cortices are part of attentional networks which are under-functioning in ADHD (Hart et al., 2013; Norman et al., 2016) (but see also (Samea et al., 2019)). ADHD-related right PFC hypoactivity is additionally manifested as reduced excitability in response to TMS directed to the rPFC (Avnit et al., 2023; Hadas et al., 2021), and as Alpha EEG asymmetric activity during resting-state (Hale et al., 2010, Hale et al., 2009; Keune et al., 2015, Keune et al., 2011), suggesting that enhancement of rPFC activity through high frequency repetitive TMS (HF rTMS), or alternatively shifting the balance of PFC activity toward the right hemisphere through an alternative stimulation protocol can improve ADHD symptoms (Keune et al., 2015).

Indeed, the potential of repetitive TMS (rTMS) as a treatment tool for ADHD has been investigated mainly by targeting the rPFC of adults using high frequency (HF) stimulation sessions over several weeks. For example, one study found that 6 weeks of HF rPFC rTMS treatment combined with atomoxetine is more effective than rTMS alone or atomoxetine alone (Cao et al., 2018). Another study (Alyagon et al., 2020) found that 3 weeks of HF rPFC deep TMS (dTMS) treatment improved ADHD symptoms and performance in an attentional task compared to both active control (treatment delivered to a non-effective stimulation area), or a sham stimulation. Another study (Bleich-Cohen et al., 2021) found that 3 weeks of HF rPFC dTMS treatment improved inattention\memory subscale (but not the overall ADHD symptoms), and increased fMRI activations in the right dorsolateral prefrontal and other right sided cortices, compared to HF left PFC and sham treatments. Nevertheless, 3 weeks of dTMS treatment directed to bi-lateral PFC was found non-effective compared to sham stimulation, possibly due to simultaneous stimulation of opposing targets (Paz et al., 2018); and another sham-controlled study failed to find significant differences between the active and sham rPFC HF rTMS groups (Weaver et al., 2012). Thus, results generally support the targeting of rPFC hypoactivity with HF rTMS, yet outcomes are inconsistent. Additional large sham-controlled trials are needed to affirm effectiveness, and individualization of treatment protocols according to clinical and brain activity parameters is warranted (Rubia et al., 2021).

A recent study identified a novel electrophysiological biomarker that highly predicts HF rPFC dTMS treatment outcome in ADHD (Alyagon et al., 2020). This was based on low-gamma and alpha EEG activity evoked by the rTMS trains during the first treatment session, which retrospectively predicted improvement of ADHD symptoms following three-weeks course of daily treatments. Importantly, this biomarker demonstrated an inverse interhemispheric association pattern with symptomatic improvement: the rTMS-evoked low-gama/alpha ratio in the rPFC was positively correlated with improvement in ADHD symptoms, while in the lPFC it was negatively correlated with improvement. The authors suggested that this biomarker reflects the immediate asymmetric response of the cortex to repetitive stimulation (i.e., right rTMS evoked excitability), and hence, we aimed here to test whether biomarker-compatible individuals are less rPFC hypoactive and suffer from milder symptoms than those who are non-compatible for rTMS treatment.

Cortico-cortical Paired Associative Stimulation (ccPAS) represents an alternative TMS approach that holds high promise for treating ADHD and other laterality-related pathologies. This bi-focal, lagged stimulation paradigm, rooted in Hebbian principles, is designed to modulate connectivity between two interconnected brain regions (Koch, 2020). Previous research indicates that a single ccPAS session targeting the left and right PFC not only enhances inter-regional connectivity but also shifts asymmetric alpha activity of healthy participants in accordance with the stimulation order. Specifically, left-then-right stimulation (LR-ccPAS) enhances left-to-right connectivity and shifts alpha activity to the right, and vice-versa for right-then-left PAS (RL-ccPAS) (Zibman et al., 2019). Therefore, the effect generated by ccPAS reflects changes in the crosstalk between the specific stimulated regions, rather than merely a stimulation-induced modulation of a target region and its less-specific downward pathways (Siebner et al., 2022), as generated by traditional rTMS. Accordingly, ccPAS may benefit individuals deemed by the biomarker as less favorable candidates for HF-rPFC dTMS by shifting interhemispheric activity balance rather than enhancing unilateral excitability. Nevertheless, ccPAS has not yet been applied to ADHD patients, and it remains unclear which order of stimulation may be most beneficial for this population.

Taken together, the present study has three main objectives: (1) To characterize the clinical profile of individuals expected to benefit from HF-rPFC dTMS (biomarker-compatible) compared with those not expected to benefit (non-compatible); (2) To prospectively initiate validation of the biomarker by comparing treatment outcomes of HF-rPFC dTMS in biomarker-compatible participants to an existing database of participants randomly assigned to HF-rPFC or sham dTMS (Alyagon et al., 2020); and (3) To explore the safety and potential efficacy of ccPAS as an alternative treatment for ADHD, by randomized allocation of non-compatible participants to either LR-ccPAS or RL-ccPAS protocols.

## 2 Methods

This double blind, semi-randomized study was approved by the institutional review board of the Soroka University Medical Center and was registered in the Israeli Ministry of Health database (MOH_2019-07-11_007266).

### 2.1 Participants

Fifty-eight TMS-naive adults with prior diagnosis of ADHD were recruited to the current study over a 4-year period through advertisements, mass university e-mails and social media. All participants signed an informed consent prior to enrolment and were screened by a senior psychiatrist using a semi structured interview (SCID) based on DSM-V criteria to verify their ADHD diagnosis and to rule out psychiatric comorbidities. Inclusion and exclusion criteria were similar to those described in our previous study (Alyagon et al., 2020) and are detailed in the supplementary methods (section 1.1).

### 2.2 Study design

To characterize the clinical profile related to biomarker-compatibility, all participants underwent a biomarker test (similar to HF dTMS treatment session) and resting-state EEG recording. Thereafter, to validate the biomarker, biomarker-compatible participants were allocated to receive HF treatment (BioA-HF) and outcomes were compared to those of an existing database of participants treated in a previous study (Alyagon et al., 2020) who were randomly allocated to HF (RA-HF) or Sham treatment (RA-Sham). Those participants were recruited using identical criteria and treated according to the same protocol (Alyagon et al., 2020), thereby providing a well-characterized reference cohort for comparison (Ghadessi et al., 2020). Finally, to initiate exploration of the safety and potential efficacy of ccPAS as an alternative treatment, non-compatible participants were randomized to receive either LR-ccPAS or RL-ccPAS protocols and were evaluated on the same timeline using the same outcome measures.

### 2.3 Procedure

Biomarker indices, computed as the power ratio between low-gamma (30–40 Hz) to alpha (8–14.5 Hz) band activity during the inter-train-interval of HF stimulation, were extracted from the EEG of all 58 subjects, as previously described (Alyagon et al., 2020) (see also supplementary methods section 1.3). Participants were denoted as biomarker-compatible if their score predicted ≥10 points improvement in the Conners' Adult ADHD Rating Scale (CAARS) total symptoms standardized score. This cutoff was chosen according to binary definition of clinical response used in the previous study (Alyagon et al., 2020), translated into a continuous improvement scale (see Fig. S1). Non-compatible participants, or those who could not endure the HF stimulation, were randomly allocated to receive either LR-ccPAS or RL-ccPAS treatment. All participants were TMS naive and not aware of their allocated treatment group.

Regardless of stimulation protocol, participants received 15 daily treatments over a course of 3 weeks (5 treatments per week), with each treatment preceded by an attention-recruiting task (Stern et al., 2016) aiming to activate the relevant brain circuitry and promote clinical response (Alyagon et al., 2020; Carmi et al., 2019; Harel et al., 2022). Clinical assessments and EEG were performed at the first treatment day (baseline; BL), last treatment day (post-treatment; Post), and at the one-month follow-up (FU) visit, which also included a maintenance treatment session.

### 2.4 TMS

HF-rPFC dTMS was administered using a Magstim Rapid^2^ stimulator (Magstim LTD., UK) equipped with an H6-coil (Alyagon et al., 2020) (Brainsway, Israel), while ccPAS was delivered using a dual channel stimulator equipped with two H—D1 coils incorporated into a single helmet (Zibman et al., 2019) (Brainsway, Israel), targeting the right and left PFC. Individual hand resting motor threshold (RMT) was measured at the beginning of each session and treatment intensity was set at 120% of RMT. For ccPAS both left and right RMT were assessed, and stimulation intensity was individually calibrated based on the respective RMT values. During treatment, the HF coil was positioned 5 cm anteriorly and 2 cm laterally to the motor hot-spot (Alyagon et al., 2020), while ccPAS coils were positioned 6 cm anteriorly to the motor hot-spot (helmet leveled) (Zibman et al., 2019). HF-rPFC stimulation included 40 trains of 18 Hz, which were 2 s long and 20 s apart (total of 1440 pulses per session) (Alyagon et al., 2020), while ccPAS included 600 paired pulses delivered 3 s apart and with an inter-pulse interval of 8 ms (Zibman et al., 2019).

### 2.5 Clinical assessment

Participants completed the CAARS self-report (long version) (Conners et al., 1999a; Erhardt et al., 1999), Behavioral Rating Inventory for Executive Functioning (BRIEF-A) (Roth et al., 2005), and Beck Depression Inventory (BDI) (Beck et al., 1961). CAARS scores were standardized according to age and gender (Conners et al., 1999b) (normal distribution, mean = 50, SD = 10).

Change in the CAARS ADHD total symptoms score following the 3 weeks of treatment was pre-defined as a primary measure for treatment outcome while other subscales of the CAARS and BRIEF-A were treated as secondary outcomes (Alyagon et al., 2020). Because the Total Symptoms score is derived from the Inattention and Hyperactivity subscales (Conners et al., 1999b), these secondary measures are reported alongside the primary outcome to aid interpretation of the specific symptom dimensions underlying changes in the Total Symptoms score.

### 2.6 Profiling of biomarker compatibility

Biomarker-compatible participants were expected to exhibit lower baseline levels of the primary measure of ADHD total symptoms and longer Stop Signal Reaction Time (SSRT), an index of response inhibition known to be impaired in ADHD (Avnit et al., 2023). In addition, following initial results, and given previous evidence linking ADHD symptoms to altered frontal alpha asymmetry (Hale et al., 2010, Hale et al., 2009; Keune et al., 2015, Keune et al., 2011), we explored the relationship between ADHD symptom severity and resting-state alpha asymmetry across the biomarker compatibility groups.

### 2.7 Biomarker validation

The biomarker was originally developed to predict improvement in total ADHD symptoms – a measure based on DSM criteria. Nevertheless, the biomarker's predictive power was even stronger for the CAARS ADHD index - a subscale comprised of the 12 most ADHD-healthy population differentiating items (Conners et al., 1999b) (Fig. S2). Consequently, both scales were used to validate the biomarker-based allocation process.

### 2.8 Stop Signal Task

The Stop signal task was delivered before and following TMS treatments as detailed in the supplementary materials (section 1.2). SSRT was computed as the average reaction time in the go trials subtracted by the average delay period of the stop signals from the go cues.

### 2.9 EEG

EEG was acquired using a TMS-compatible 64-channels amplifier (TMSi Ltd.) and cap (ANT Ltd.), with Cz as a common reference (re-referenced offline to the average), and POz as the ground. Resting-state data was acquired for 5 min with eyes closed. Data was preprocessed using EEGlab (Delorme and Makeig, 2004) and custom scripts in Matlab (R2023a; see supplementary methods section 1.3), and clean data was transformed into the frequency domain using the Welch's method. Asymmetry scores were then computed as the power ratio between alpha activity in each electrode and its homologous interhemispheric pair. To stabilize asymmetry maps (Zangen et al., 2023), observations deviating by 2.5 standard scores or more (29 out of 1568 observations = 1.8%), were channel-wise replaced by the mean.

### 2.10 Statistical analysis

Statistical analysis of clinical data was conducted using STATISTICA software (v 14.0.1.25; TIBCO Software Inc.) and Matlab. All statistical inference was performed using two tailed tests requiring an a-priory alpha level of 5%, unless detailed otherwise.

Biomarker allocation-related improvement in CAARS, BDI and BRIEF-A scales, controlled for the influence of baseline levels, were estimated using an ANCOVA model with treatment group (BioA-HF, RA-HF, RA-Sham) as between-subjects' factor and baseline score as a nested covariant (Barnett et al., 2005). Time points (Post, FU) were evaluated separately to avoid observations lost due to missing values of participants who reached the primary end point (Post-treatment) but did not show up for the FU visit.

As noted above, the biomarker was previously suggested to be inversely associated with right PFC activation and with ADHD severity (Alyagon et al., 2020), and hence biomarker-compatible participants were expected to demonstrate both lower baseline symptoms and greater beneficial treatment outcome compared to all other groups. These a-priory hypotheses were therefore tested using one-tailed contrasts, and other post-hoc tests (with the sham group) were avoided. Additionally, the analyses were guided by prespecified hypotheses and a hierarchical outcome framework that distinguished primary and secondary outcome measures. Consistent with this predefined analytic strategy, no correction for multiple testing was applied (Althouse, 2016; Parker and Weir, 2022).

The influence of ccPAS on treatment outcomes was evaluated using repeated measures ANOVA with PAS order (LR, RL) as between-subjects' factor, and time (baseline, post-treatment, FU) as within-subjects' factor. As described above, time points were separately evaluated to avoid loss of observations and baseline symptom levels were additionally controlled for using ANCOVA models.

Significant allocation- or treatment-related effects were further subjected to sensitivity analysis using linear mixed-effects models. These models incorporate all available repeated measurements and account for incomplete follow-up under a missing-at-random assumption (see details in supplementary methods section 1.4).

Correlation between resting-state asymmetry and baseline ADHD symptom levels was explored using non-parametric permutation analysis (Monte-Carlo method) implemented in FieldTrip (Oostenveld et al., 2011). Multiple tests due to electrodes number were corrected using a cluster-based approach.

## 3 Results

Fifty-eight participants were recruited to the experiment and underwent a biomarker test for HF-compatibility. For clinical profiling of biomarker-defined responders, 24 were found to be biomarker-compatible and 32 non-compatible (Table 1A), while 2 had not completed the compatibility test. For the biomarker initial validation arm of this study, 15 biomarker-compatible participants, who were willing to commit to the extended treatment and follow-up protocol, were assigned to receive HF-rPFC dTMS (BioA-HF) treatment. Their outcomes were compared to those previously randomized to active (RA-HF; *n* = 15) or sham (RA-Sham; *n* = 14) treatment (Table 1B). For the exploration on safety and potential efficacy of ccPAS as an alternative treatment, 24 non-compatible participants were randomly allocated (1:1) to either the RL-ccPAS treatment arm or the LR-ccPAS treatment arm (Table 1C). Two participants of the BioA-HF group and 1 of the RL-ccPAS group discontinued treatment before reaching endpoint and were excluded from the analysis. Additionally, 1, 4, 1, 1, and 2 participants completed the treatment but were lost to follow-up in the BioA-HF, RA-HF, RA-Sham, LR-ccPAS and RL-ccPAS respectively. The most common reason for loss to follow-up was scheduling difficulties. One participant cited the burden associated with the EEG assessment, while two participants did not provide a reason for non-attendance. The treatment groups did not differ in their demographic profile.

**Table 1:** Demographic profile. Demographic, clinical and behavioral baseline characteristics of the compatibility (A) HF (B) and ccPAS (C) groups. Note that compatibility-related differences in ADHD symptoms and SSRT are reported in the next sections. BDI: Back Depression Inventory; BRIEF-A GEC: Behavioral Rating Inventory for Executive Functioning – Global Executive Composite; ADHD Total Sympt: Conners' Adult ADHD Rating Scale – Total Symptoms score; SSRT: Stop Signal Reaction Time.

A	HF Compatible	HF Non-Compatible	p
F/M	8/16	9/23	0.67
Age ± SD	25.5 ± 3.0	27.7 ± 5.2	0.07
BDI ± SD	6.5 ± 5.3	4.7 ± 4.9	0.21
BRIEF-A GEC ± SD	64.5 ± 9.9	72.1 ± 21.8	0.14


B	Bio-HF	RA-HF	RA-Sham	p
F/M	3/10	2/13	3/11	0.55
Age ± SD	26.4 ± 2.5	26.6 ± 5.9	27.6 ± 2.3	0.23
BDI ± SD	4.6 ± 3.9	6.7 ± 2.8	8.36 ± 8.1	0.21
BRIEF-A GEC ± SD	63.7 ± 11.6	69.4 ± 7.8	67.4 ± 11.4	0.35


C	LR-ccPAS	RL-ccPAS	p
F/M	4/8	3/8	0.75
Age ± SD	27.7 ± 6.1	27.8 ± 3.6	0.97
ADHD Total Sympt ± SD	77.0 ± 8.3	75.1 ± 8.1	0.58
BDI ± SD	6.0 ± 4.1	5.6 ± 4.5	0.84
BRIEF-A GEC ± SD	68.2 ± 9.8	69.3 ± 8.0	0.79
SSRT ± SD	296.0 ± 46.1	315.5 ± 40.7	0.31

### 3.1 Clinical profiling of biomarker-defined responders

CARRS total ADHD score of biomarker-compatible participants was significantly lower than that of the non-compatible individuals (t_(54)_ = 1.699, *p* = 0.047, d_Cohen_ = 0.46). This result was driven by significantly reduced hyperactivity symptom score (t_(54)_ = 1.88, *p* = 0.032, d_Cohen_ = 0.51) in biomarker-compatible participants, while inattention symptom score did not differ between groups (t_(54)_ = 0.657, *p* = 0.25; Fig. 1A). Additionally, the stopping time (SSRT) of biomarker-compatible participants was significantly shorter than that of non-compatible participants (t_(47)_ = 1.758, *p* = 0.042, d_Cohen_ = 0.53; Fig. 1B).

**Fig. 1:**
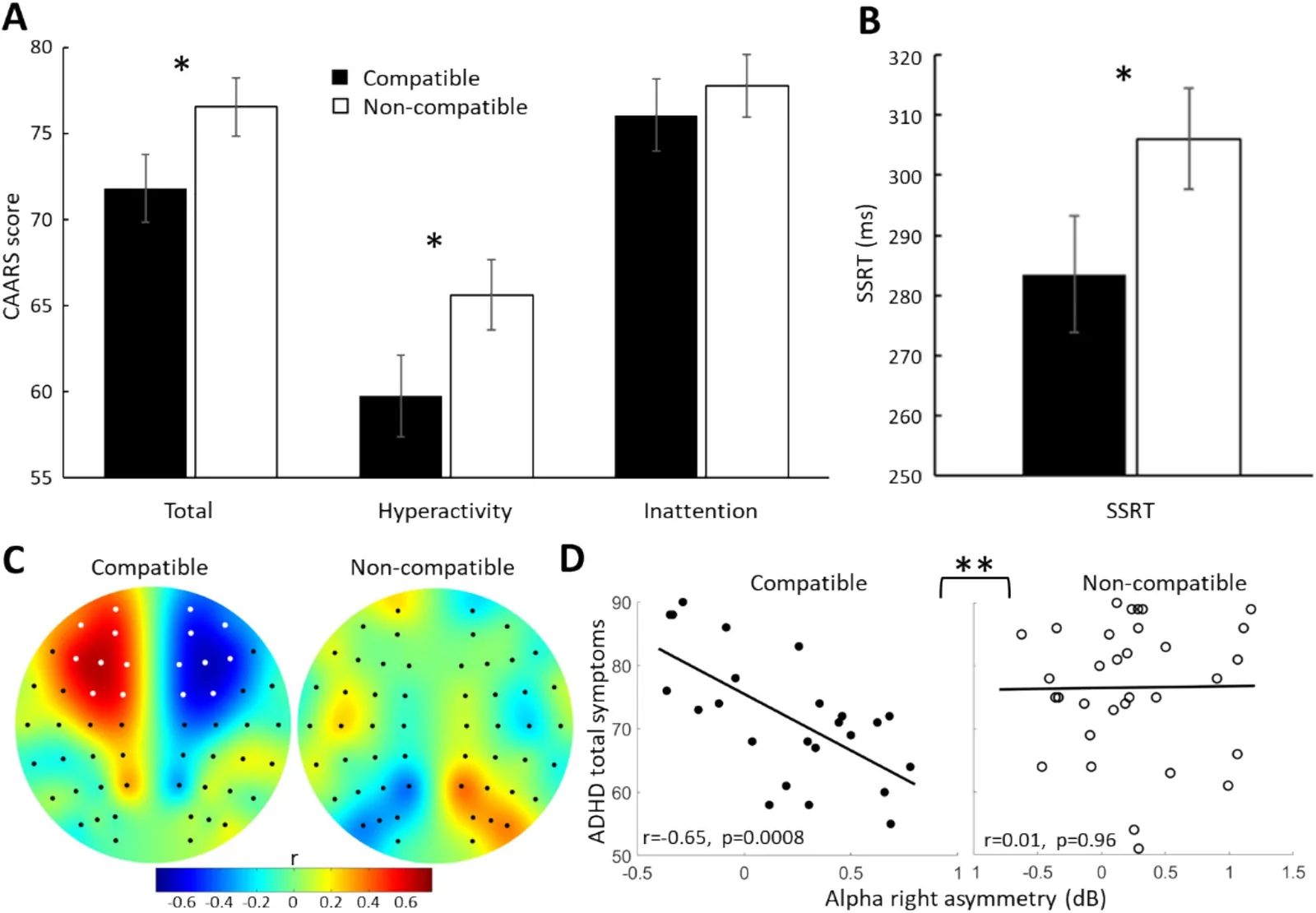
Influence of biomarker-allocation on baseline level of ADHD symptoms. (A) Bar graph of inattention, hyperactivity and total ADHD baseline symptoms (mean ± SEM) measured by the CAARS questionnaire for biomarker-compatible and non-compatible participants. (B) Same for SSRT. (C) Topographical plots of the correlation magnitude between resting-state alpha asymmetry and CAARS total ADHD baseline symptoms for the two groups. Electrodes indicating significant correlation are marked white. (D) Scatter plots for this correlation at rPFC electrode F4. *p < 0.05, **p < 0.01.

We then further explored whether these differences in baseline ADHD symptom levels were related to asymmetric resting-state activity in the alpha band, as previously suggested (Hale et al., 2010, Hale et al., 2009; Keune et al., 2015, Keune et al., 2011). Non-parametric permutation test indicated a significant negative correlation between total ADHD symptoms and alpha right asymmetry scores in a cluster of 8 fronto-lateral electrodes within the biomarker-compatible group (*p* = 0.0056), peaking at electrode F4 (above dorsolateral-prefrontal cortex; r_(*n*_ _=24)_ = −0.65, *p* = 0.0008; Fig. 1C and D). This pattern was not evident in HF non-compatible participants (r_(*n*_ _=32)_ = 0.013, *p* = 0.96), or in the entire sample (r_(*n*_ _=58)_ = −0.21, *p* = 0.12; not shown) and correlation magnitude differed between the compatibility groups (Z_Fisher_ = 2.75, *p* = 0.003, q_Cohen_ = 0.79).

### 3.2 Biomarker validation

In a similar manner to the above, CAARS total ADHD symptom and index levels in the biomarker-compatible group were significantly lower than those of the RA-HF group (total symptoms: t_(26)_ = 3.07, *p* = 0.004, d_Cohen_ = 1.17; index: t_(26)_ = 1.82, *p* = 0.08, d_Cohen_ = 0.69) or the RA-Sham group (t_(25)_ = 2.18, *p* = 0.03, d_Cohen_ = 0.84; t_(25)_ = 2.34, *p* = 0.02, d_Cohen_ = 0.90), introducing an inherent difficulty to test the contribution of the allocation process to treatment outcome due to possible regression to the mean problem (Fig. 2A; only ADHD total symptoms are shown. See also Fig. S3 for individual trajectories). Furthermore, while a strong positive correlation between baseline and post-treatment ADHD symptom scores was observed in the RA-Sham group (*r* = 0.93, *p* < 0.0001), a lower correlation was shown in the RA-HF group (*r* = 0.69, *p* = 0.004), and no significant correlation was found in the BioA-HF group (*r* = 0.29, *p* = 0.33; Fig. 2C). This suggests that treatment reduces the tight correlation between baseline and post-treatment scores in both active stimulation groups, but to a greater extent in the BioA-HF group.

**Fig. 2:**
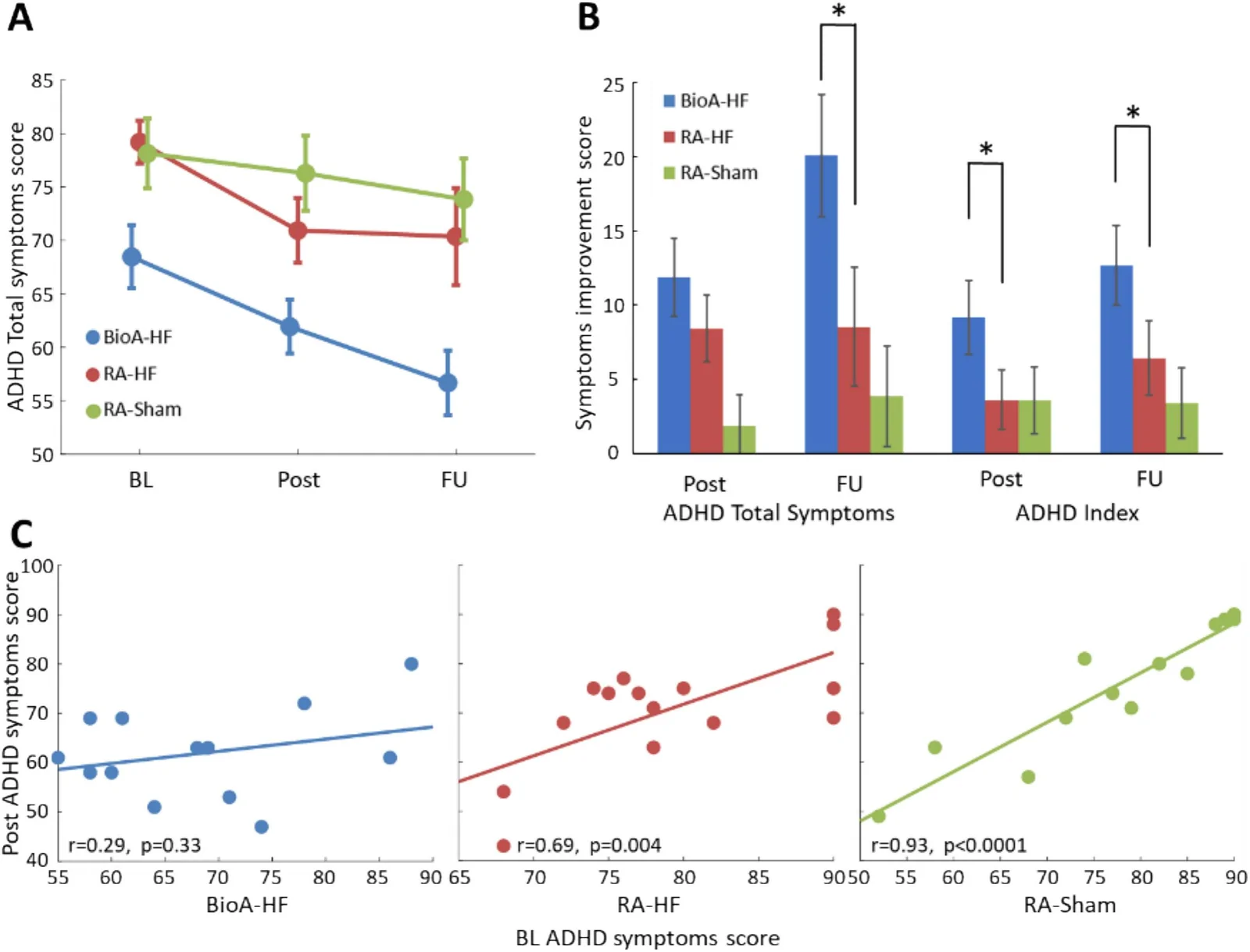
Influence of allocation on HF treatment outcome. (A) Line plot showing the influence of allocation (BioA-HF, RA-HF, RA-Sham) on CAARS ADHD total symptoms (mean ± SEM) across baseline (BL), post-treatment (Post) and follow-up (FU) time points. (B) Bar plots showing estimated marginal means (±SEM) from ANCOVA models for improvement in ADHD total symptoms and ADHD index adjusted for baseline level. (C) Scatter plots depicting differential correlations between baseline and post-treatment ADHD total scores across allocation groups. *p < 0.05.

To mitigate this regression to the mean problem, we employed repeated measures ANCOVA models to evaluate treatment improvement controlled for the influence of baseline levels (Barnett et al., 2005) (see statistical methods and Fig. 2B). Significant treatment Group X Covariant interaction effects were observed for both scales (total symptoms: F_(3, 36)_ = 4.28, *p* = 0.01, η^2^_partial_ = 0.26; ADHD index: F_(3, 36)_ = 4.74, *p* = 0.007, η^2^_partial_ = 0.28), confirming that the association between baseline levels and clinical outcome differed among the treatment groups. Model estimated means of improvement were consistently highest in the BioA-HF group, followed by the RA-HF group and lowest in the RA-Sham group across both scales at post-treatment and FU (Supplementary Table S1). Planned comparisons revealed that improvement in the BioA-HF group was significantly greater than that of the RA-HF group at both time points for the ADHD index (Post-treatment: F_(1, 36)_ = 3.04, *p* = 0.045, η^2^_partial_ = 0.08; FU: F_(1,30)_ = 2.86, *p* = 0.05, η^2^_partial_ = 0.09) and for the ADHD total symptoms only at FU (Post- treatment: F_(1, 36)_ = 0.98, *p* = 0.16, η^2^_partial_ = 0.027; FU: F_(1, 30)_ = 4.05, *p* = 0.027, η^2^_partial_ = 0.12). Sensitivity analysis conducted using a mixed-effects regression model confirmed these findings, suggesting that allocation influence is robust to missing follow-up data points and is unlikely to be driven by attrition (supplementary tables S2, S3). Other CAARS and BRIEF-A subscales showed similar (but non-significant) trends (Fig. S4).

### 3.3 Safety and efficacy of ccPAS treatment

No adverse events were reported other than mild and transient headaches and scalp discomfort localized to the frontal stimulation area.

The repeated measures ANOVA indicated a reduction of ADHD total symptoms in the two groups following treatment (F_(1,21)_ = 6.61, *p* = 0.018, η^2^_partial_ = 0.24), yet no Order X Time interaction was evident (F_(1, 21)_ = 1.94, *p* = 0.18, η^2^_partial_ = 0.08; Fig. 3, S5). Dividing the ADHD symptoms to their subscales yielded a trend of enhanced reduction in hyperactivity-impulsivity symptoms in the LR-ccPAS group relative to the RL-ccPAS group (F_(1, 21)_ = 3.24, *p* = 0.086, η^2^_partial_ = 0.13), but such trend was not maintained in FU (F_(1, 18)_ = 0.50, *p* = 0.49, η^2^_partial_ = 0.03). Descriptively, the magnitude of improvement observed following ccPAS appeared smaller than that seen in the BioA-HF group (shown for comparison in Fig. 3; see also Table S4), although this group was not included in the present analysis. Investigation of other secondary measures yielded no significant results. (Fig. S6).

**Fig. 3:**
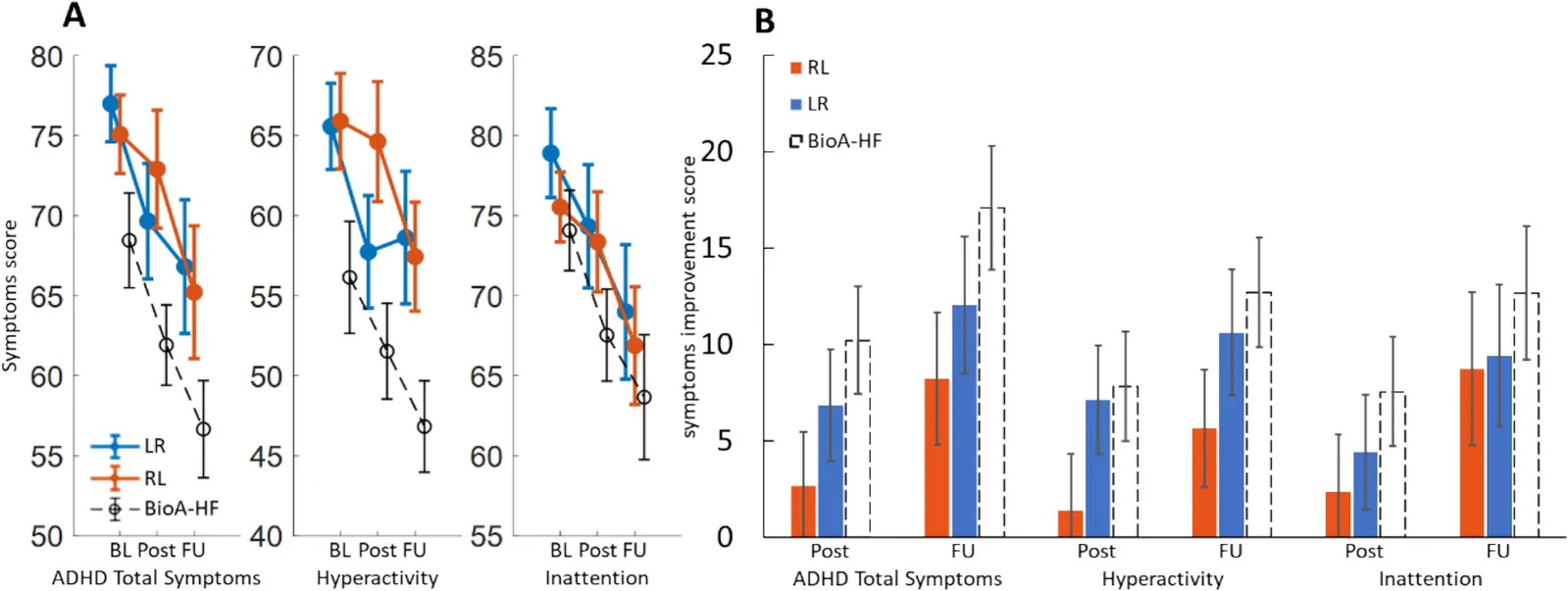
Influence of ccPAS treatment on ADHD symptoms. Line plots indicating the influence of ccPAS order (LR, RL) on CAARS inattention, hyperactivity and total ADHD scores (mean ± SEM) across baseline (BL), post-treatment (Post) and follow-up (FU) time points. The Bio-HF group is included for comparison. (B) Bar plots showing estimated means (±SEM) from the ANCOVA models for improvement in hyperactivity, inattention and total ADHD symptoms adjusted for baseline level.

## 4 Discussion

The present study demonstrates the ability of a previously-described TMS-EEG low-gamma/alpha biomarker to prospectively classify participants with ADHD into subpopulations exhibiting different clinical characteristics. Participants who had high levels of the rTMS-evoked low-gamma to alpha activity ratio under the stimulation area, and thus were classified as compatible for HF treatment, displayed distinct characteristics compared to non-compatible participants. These include lower baseline level of ADHD-related clinical symptoms, particularly hyperactivity symptoms, as well as enhanced behavioral inhibition (shorter SSRT). In addition, this subgroup demonstrated more favorable treatment outcomes, when compared to those who were randomly allocated to receive active treatment without any biomarker selection. The subpopulation that was not expected to benefit from HF-rPFC dTMS and therefore allocated to the exploratory ccPAS intervention, showed only modest improvements in symptoms, which were not sensitive to the order of pulses (right to left versus left to right). Consistent with previous evidence (Avnit et al., 2023; Hadas et al., 2021; Hale et al., 2010, Hale et al., 2009; Hart et al., 2013; Keune et al., 2011; Norman et al., 2016), these findings further support right prefrontal hypoactivity as an etiological factor in ADHD, by demonstrating the inverse relationship between brain rTMS evoked excitability and the level of clinical and behavioral symptoms, and between evoked excitability and the propensity to respond to brain stimulation.

More broadly, these results contribute to ongoing efforts to develop TMS-EEG-derived biomarkers (Ziemann et al., 2026), particularly studies combining electrophysiological measures with cognitive or behavioral phenotyping (Di Fazio and Palermo, 2026; Zangen et al., 2023) with both diagnostic and prognostic utility, enabling the identification of biologically meaningful patient subgroups, the prediction of treatment responsiveness, and the tracking of neurophysiological changes associated with therapeutic interventions.

The discriminative ability of the biomarker is further supported by the differing relationship between prefrontal asymmetric activity at rest and symptom levels observed in the two groups. Specifically, a negative relationship between right hemispheric asymmetric activity in the alpha band and the overall ADHD symptom level was observed in biomarker-compatible but not in non-compatible participants. The negative direction of this association is unique to biomarker-compatible patients, as it was not observed in two entire cohorts of individuals with ADHD collected in our lab, both in the current and a previous study (Avnit et al., 2023). In those cohorts, however, a relationship between ADHD symptoms and right hemispheric hypoactivity was demonstrated when the latter was measured in response to magnetic stimulation (single or repetitive pulses), highlighting the advantage of measuring brain activity level using active rather than passive means. Moreover, this negative direction of association contradicts existing literature (Hale et al., 2010, Hale et al., 2009; Keune et al., 2015, Keune et al., 2011) and may explain conflicting findings in the field, which did not include such subpopulation division (Alperin et al., 2019; Baving et al., 1999; Gordon et al., 2010; Jaworska et al., 2013). The distinct profile of the groups also suggests that future biomarkers for response to treatment can be constructed based on the clinical profile alone, or clinical profile together with resting-state EEG, and thus to reduce the burden of undergoing the HF dTMS treatment session to establish compatibility.

As the biomarker itself relies on asymmetric activity evoked by a unilateral stimulation, the difference between the biomarker-compatible and non-compatible subpopulations may reflect structural or functional alterations in frontal inter- or intra hemispheric pathways, which are known to be compromised in ADHD patients (Albajara Sáenz et al., 2019; Parlatini et al., 2023; Yu et al., 2023) and particularly in the combined type compared to primarily inattentive disorder (Wu et al., 2022). Given their more inattentive and less hyperactive symptoms' profile, future studies should investigate if HF-compatible participants also possess better-preserved interhemispheric pathways, explaining the tighter relationship observed between their symptom levels and resting asymmetric activity.

Investigation of the influence of biomarker-based allocation on treatment efficacy revealed that BioA-HF participants exhibited greater improvement in overall CAARS ADHD symptoms level compared to the RA-HF group at the one-month follow-up, but not immediately at post-treatment. For the ADHD index, which comprises the twelve items that most effectively differentiate between individuals with ADHD and healthy controls (Conners et al., 1999b), BioA-HF participants had enhanced improvement at both time points. These findings suggest that the biomarker may predict a therapeutic improvement in the core symptoms of ADHD, with secondary symptoms alleviated as a downstream effect. The magnitude of the allocation-related improvement was in the medium-to-large range (partial η^2^ = 0.08–0.12), which is encouraging given the complexity and heterogeneity of ADHD and the fact that treatment response was predicted solely by an electrophysiological assessment. At the same time, these effect sizes indicate that biomarker-guided allocation does not fully account for individual variability in treatment outcome and therefore leaves room for further refinement of treatment prediction. Our ability to demonstrate enhanced therapeutic outcome of biomarker-allocation was compromised due to the lower baseline symptoms level of that treatment group, causing regression to the mean and underestimation of the therapeutic response. Inclusion of baseline symptom levels as a covariate in future models may prevent this distortion and improve prediction.

The ccPAS treatment was found to be safe, with no unusual side effects reported. Clinically, a modest decrease in ADHD total symptom levels was observed in both treatment groups, with no significant effect of stimulation order. Although a trend toward greater improvement in the LR-ccPAS group compared to RL-ccPAS group was observed at post-treatment, this difference was not maintained at FU. Thus, the present findings do not support the hypothesis that the proposed associative mechanism of ccPAS, indexed by stimulation order, contributes meaningfully to clinical outcome in this sample. Furthermore, in the absence of a sham-control group, it remains unclear whether the modest improvement observed in both groups reflects a specific effect of bilateral prefrontal stimulation or non-specific factors such as placebo effects.

One strength of this study is that it is the first to prospectively support the biomarker's ability to predict clinical response through patient allocation to the treatment group. Notably, the same biomarker was also shown to differentiate between depressed patients more likely to respond to different TMS coils targeting the lPFC and medial prefrontal cortex in a multi-center study involving six weeks of HF-rTMS (Zangen et al., 2023). Importantly, in that study, the biomarker correlated the clinical outcome in a target-specific manner: treatment outcome following lateral prefrontal stimulation was associated primarily with the asymmetry component of the biomarker, whereas outcome following medial prefrontal stimulation was associated with its absolute magnitude. Together, these studies suggest that although related to baseline characteristics, the biomarker is pathology-independent and sensitive to the stimulation target. It may therefore reflect cortical excitability as a more fundamental physiological property rather than a marker of a specific disease phenotype.

A major limitation in this study, regarding the clinical prediction aspect of the biomarker, is that outcomes in the BioA-HF group were compared to those of a cohort randomly assigned to Active or Sham treatments in a previous study. This historical control design inherently limits internal validity, particularly in light of the substantial baseline symptom differences observed between cohorts. Accordingly, selection-related differences between cohorts, recruitment-period effects, participant expectations, staff interactions, and other unmeasured temporal factors cannot be fully excluded. Nevertheless, consistent with recommendations for usage of historical control design (Ghadessi et al., 2020), both cohorts were recruited in the same lab, using identical inclusion/exclusion criteria, identical HF dTMS intervention protocol, same outcome measure, and under the supervision and guidance of the same senior team, thereby reducing potential sources of bias. A major limitation of the ccPAS experiment is that outcomes were explored only in a subgroup of (non-compatible) ADHD participants, thus limiting generalization of results. The possibility that ccPAS could be beneficial in the general adult ADHD population cannot be ruled out, as participants who are non-compatible with HF stimulation due to low rTMS responsiveness may also be generally less responsive to electromagnetic stimulation of any protocol. Finally, the limited sample size within each subgroup renders this study exploratory rather than conclusive. Consequently, effect size estimates should be interpreted with caution, and both positive and negative findings require replication in larger independent samples. This consideration applies broadly across the study, including the clinical, biomarker, and neurophysiological analyses. Taken together, these limitations suggest that findings should be interpreted as an initial proof-of-concept evaluation of prospective biomarker-guided treatment allocation, and as a limited exploration of feasibility and safety of ccPAS treatment protocol, rather than definitive evidence of their clinical utility.

To conclude, the current study provides further evidence that within-treatment rTMS-evoked excitability, as measured by EEG, can serve as both a biomarker for symptom severity and a predictor of HF-dTMS treatment efficacy. Consequently, a large-scale validation study, directly comparing biomarker-guided allocation with conventional random allocation, is warranted to establish this biomarker as a patient-selection tool for ADHD. Additionally, while our findings suggest that prefrontal ccPAS is a safe intervention for ADHD, its therapeutic efficacy must be rigorously evaluated in a comprehensive, randomized, sham-controlled trial.

## Declaration of generative AI usage

The authors used Microsoft Copilot to improve the clarity and readability of the manuscript. No AI tools were used for data analysis, interpretation, or generation of scientific content. The authors reviewed and approved all final content.

## CRediT authorship contribution statement

**Uri Alyagon:** Writing – review & editing, Writing – original draft, Methodology, Formal analysis, Conceptualization. **Lihi Bokovza:** Writing – original draft, Project administration, Methodology, Investigation, Formal analysis. **Hadar Shalev:** Supervision, Investigation. **Noam Ygael:** Writing – review & editing. **Abraham Zangen:** Writing – review & editing, Methodology, Funding acquisition, Conceptualization.

## Ethics declaration

Written informed consent to take part in the study and to publish the article has been obtained from all participants or their legal representatives. The privacy rights of participants have been observed.

This study was performed in compliance with relevant laws, regulatory frameworks and guidelines where the research took place.

This study was approved by the IRB of Soroka Clinical Research Center.

(Approval No. 0225-19-SOR)

The results of this clinical trial and any associated work have been posted in a registry.

This clinical trial was registered with number MOH_2019-07-11_007266 (Israel Ministry of Health Clinical Trials Registry (MyTrial)).

## Funding

This research was supported by the 10.13039/501100003977Israel Science Foundation (grants No. 2079/18, 2278/22).

## Declaration of competing interest

AZ is a consultant for, and has financial interest in, Brainsway Ltd.; a company that develops transcranial magnetic stimulation (TMS) coils designed for stimulation of deeper brain areas. UA is an EEG consultant for Brainsway Ltd.

## Data Availability

Data will be made available on request.
