# Supplementary material for: Toward precision neuromodulation in ADHD: rTMS-evoked excitability as a biomarker and ccPAS as an alternative protocol
Source: Neuroimage Clin. 2026 Sep 24;52:104067. doi: 10.1016/j.nicl.2026.104067 (PMC13651681; doi:10.1016/j.nicl.2026.104067)
Supplement: Supplementary file 1 [file mmc1.docx]

Supplementary materials for

**Toward precision neuromodulation in ADHD: rTMS-evoked excitability as a biomarker and ccPAS as an alternative protocol**

Uri Alyagon^1^*, Lihi Bokovza^1^*, Hadar Shalev^2^, Noam Ygael^1^, Abraham Zangen^1^

^1^Department of Life Sciences and the Zelman Center for Brain Science Research, Ben-Gurion University of the Negev, Beer-Sheva, Israel

^2^Psychiatry Department, Soroka Medical Center, Beer-Sheva, Israel

^*^Equal contribution

Corresponding author: Prof. Abraham Zangen, Department of Life Sciences and the Zelman Center for Brain Science Research, Ben-Gurion University of the Negev, P.O. Box 653, Beer-Sheva 8410501, Israel. Tel. 972-8-6472646, Fax 972-8-6461713, E-Mail: [azangen@bgu.ac.il](mailto:azangen@bgu.ac.il)

1. **Supplementary methods**
   1.1. Criteria for participation

*Inclusion criteria****:*** (1) Age 18–65 years old; (2) ADHD diagnosis according to DSM-V; (3) consent to abstain from other treatments (e.g., methylphenidate) for 4 weeks, starting 1 week prior to the first treatment and ending after the last treatment; (4) satisfactory responses on the TMS safety questionnaire; (5) provided informed consent to participate in the study.

*Exclusion criteria:* (1) Additional axis 1 psychiatric disorder according to DSM-V; (2) treatment by antipsychotics, antidepressants, or mood stabilizers; (3) known intolerance to TMS; (4) cognitive or functional disability, diagnosed according to DSM-V criteria; (5) high risk for suicidality as assessed during the screening interview; (6) suffering from an unstable physical disease, such as high blood pressure (> 150 mmHg systolic/> 110 mmHg diastolic) or acute, unstable cardiac disease; (7) history of epilepsy or seizures – for the patient or for first degree relatives; (8) history of a head injury or cerebral infraction that caused irreversible damage; (9) history of any metal in the head (outside the mouth); (10) metallic particles in the eye, implanted cardiac pacemaker or any intracardiac lines, implanted neurostimulators, intracranial implant (e.g., aneurysm clips, shunts, stimulators, cochlear implants, or electrodes), or implanted medical pumps; (11) alcohol or other substance abuse or dependence during the last 12 months before recruitment; (12) inability to communicate reliably with the investigator; (13) participation in a clinical trial during this clinical trial or within the past 3 months before the beginning of this clinical trial; (14) inability to sign an informed consent form; (15) known or suspected pregnancy.

1.2. Stop Signal Task

A modified version of the visual stop signal task was delivered using E-prime (PST Inc., E-Prime 2.0). Participants had to respond to visual cues (‘x’, ‘o’) by pressing one of two corresponding buttons using either their left or right index fingers. In 25% of trials, a stop signal (white square) was presented immediately after the visual cue, instructing the participants to withhold their response. The stop signal delay (latency) period from visual cue onset changed in a staircase dynamic-tracking manner, depending on performance (Berger et al., 2013; Hadar et al., 2017).

1.3. EEG

EEG was acquired using a TMS-compatible 64-channels amplifier (TMSi Ltd.) and cap (ANT Ltd.) with Cz as a common reference (re-referenced offline to the average) and POz as the ground. Impedance was kept below 10 kOhm and signal was digitized at 2048 Hz using a 24-bit AD converter.

Biomarker data was extracted from brain activity during HF stimulation. Data was filtered (1-100 Hz bandpass, 48-52 Hz notch FIR filter) and 2 second segments, starting 1 second after the end of each train, were extracted from the inter-train-intervals (Alyagon et al., 2020). Data was then manually scanned and noisy channels or epochs were excluded. Eye movements and residual TMS-related artifacts were removed using ICA (Independent Components Analysis) (Rogasch et al., 2014), and power spectral density was then computed using Welch's method. The final frequency analysis was based on an average of 36.02 segments (SD=4.07) per treatment recording. Biomarker indices were computed as the ratio between low-gamma (30-40 Hz) and alpha (8-14.5 Hz) band activity.

Resting-state EEG was recorded for 5 minutes (eyes closed; 30 initial seconds were truncated to prevent state transitional influence). The pre-processing of the resting-state data was similar to that of the treatment, except that ICA was used only when excessive eye movements were apparent. This strategy is preferred over ICA components' deletion when data is abundant (Jung et al., 2000). The final frequency analysis was based on an average of 116.1 segments (SD=16.34) per resting-state recording.

1.4. Sensitivity analysis

Significant allocation-related effects were further subjected to sensitivity analysis using linear mixed-effects models. These models incorporate all available repeated measurements and account for incomplete follow-up under a missing-at-random assumption. Time, allocation, and their interaction were entered as fixed effects, together with baseline symptom level and its interaction with group. Categorical predictors were effect-coded (sum-to-zero coding), such that parameter estimates represent deviations from the grand mean rather than comparisons with a designated reference category. A random intercept was specified for each participant to account for within-subject dependence across repeated observations.

**Figure S1: Translation of binary clinical response definition to continuous improvement scale.** In the previous study, treatment response was defined as a ≥25% reduction in ADHD symptoms (Alyagon et al., 2020). To translate this predefined clinical response criterion into the continuous CAARS outcome scale used by the biomarker, we identified the minimum improvement associated with meeting the responder criterion across the cohort. Participants fulfilling the responder criterion exhibited an improvement of at least 10 standardized CAARS points, and this value was therefore prospectively adopted as the threshold for treatment allocation in the current study.

**Figure S2: Comparison between ADHD total symptoms and ADHD index as outcome measures for biomarker-based prediction.** (A) Topographical plots of the correlation between the rTMS-evoked biomarker (low-gamma/alpha band asymmetric activity) and the CAARS ADHD total symptoms and ADHD index scales, **as previously measured in the RA-HF cohort** (Alyagon et al., 2020). (B) Scatter plot of this correlation at right prefrontal electrode FC4. Although the ADHD total symptoms scale was chosen as an outcome measure for its relevance to DSM criteria, the biomarker’s predictive power was even stronger for the ADHD index.

2. **Supplementary results**

Supplementary Tables

Table S1

|  |  | Allocation Group | | |
| --- | --- | --- | --- | --- |
| Scale | Time | BioA-HF | RA-HF | RA-Sham |
| ADHD Total Symptoms EMM [95% CI] | Post | 11.86 [6.54, 17.18] | 8.45 [3.90, 12.99] | 1.87 [-2.45, 6.19] |
|  | FU | 20.07 [11.69, 28.46] | 8.54 [0.37, 16.70] | 3.87 [-3.01, 10.75] |
| ADHD Index EMM [95% CI] | Post | 9.17 [4.18, 14.17] | 3.63 [-0.45, 7.70] | 3.61 [-0.95, 8.16] |
|  | FU | 12.67 [7.18, 18.16] | 6.44 [1.29, 11.59] | 3.41 [-1.47, 8.28] |

**Table S1:** Estimated marginal means (EMMs) and 95% confidence intervals (CIs) from ANCOVA models evaluating improvement in ADHD Total Symptoms and ADHD Index scores across allocation groups at post-treatment (Post) and follow-up (FU), adjusted for baseline symptom severity.

Table S2

**A**

| ANOVA | | |
| --- | --- | --- |
| Term | F_(1, 69)_ | p |
| Time | 3.35 | 0.0717 |
| BL | 9.08 | 0.0036 |
|  | F_(2, 69)_ |  |
| Treatment | 7.03 | 0.0017 |
| BL X Treatment | 4.89 | 0.0103 |
| Time X Treatment | 1.04 | 0.3601 |

**B**

| Model Coefficients | | | |
| --- | --- | --- | --- |
| Term | b | t(69) | p |
| Intercept | 8.86 | 6.21 | 0.0000 |
| Time: FU | 1.29 | 1.83 | 0.0717 |
| Treatment: BioA-HF | 7.08 | 3.32 | 0.0014 |
| Treatment: RA-HF | -0.92 | 0.46 | 0.6477 |
| BL | 0.40 | 3.01 | 0.0036 |
| BL X Treatment: BioA-HF | 0.56 | 3.05 | 0.0033 |
| BL X Treatment: RA-HF | -0.29 | 1.38 | 0.1723 |
| Time: FU X Treatment: BioA-HF | 1.31 | 1.31 | 0.1931 |
| Time: FU X Treatment: RA-HF | -1.20 | 1.19 | 0.2380 |

**C**

| BioA - RA Planned Contrast | | |
| --- | --- | --- |
| Time | F_(1, 69)_ | p(one-tailed) |
| Post | 1.94 | 0.0839 |
| FU | 6.35 | 0.007 |

**Table S2: Sensitivity analysis of allocation effects on improvement in CAARS ADHD total symptoms.** (A) ANOVA-like tests of fixed effects. (B) Fixed-effect parameter estimates. (C) Planned one-tailed contrasts comparing improvement in the BioA-HF and RA-HF groups at post-treatment and follow-up (FU). Results replicated the primary analysis, demonstrating greater improvement in the BioA-HF group at follow-up but not at post-treatment and supporting the robustness of the allocation effect to missing follow-up data.

Table S3

| ANOVA | | |
| --- | --- | --- |
| Term | F_(1, 69)_ | p |
| Time | 2.30 | 0.1339 |
| BL | 14.92 | 0.0003 |
|  | F_(2, 69)_ |  |
| Treatment | 3.96 | 0.0236 |
| BL X Treatment | 5.37 | 0.0068 |
| Time X Treatment | 1.15 | 0.3211 |

| Model Coefficients | | | |
| --- | --- | --- | --- |
| Term | b | t_(69)_ | p |
| Intercept | 6.42 | 5.77 | 0.0000 |
| Time: FU | 0.76 | 1.52 | 0.1339 |
| Treatment: BioA-HF | 4.59 | 2.78 | 0.0070 |
| Treatment: RA-HF | -1.59 | 1.06 | 0.2935 |
| BL | 0.55 | 3.86 | 0.0003 |
| BL X Treatment: BioA-HF | 0.18 | 0.98 | 0.3301 |
| BL X Treatment: RA-HF | 0.36 | 1.49 | 0.1413 |
| Time: FU X Treatment: BioA-HF | 0.76 | 1.06 | 0.2910 |
| Time: FU X Treatment: RA-HF | 0.26 | 0.36 | 0.7182 |

| BioA - RA Planned Contrast | | |
| --- | --- | --- |
| Time | F_(1, 69)_ | p(one-tailed) |
| Post | 3.74 | 0.0285 |
| FU | 4.65 | 0.0172 |

**Table S3: Sensitivity analysis of allocation effects on improvement in CAARS ADHD index.** (A) ANOVA-like tests of fixed effects. (B) Fixed-effect parameter estimates. (C) Planned one-tailed contrasts comparing improvement in the BioA-HF and RA-HF groups at post-treatment and follow-up (FU). Results replicated the primary analysis, demonstrating greater improvement in the BioA-HF group at both post-treatment and follow-up, supporting the robustness of the allocation effect to missing follow-up data.

Table S4

|  |  | ccPAS order | |
| --- | --- | --- | --- |
| Scale | Time | RL | LR |
| ADHD Total Symptoms EMM [95% CI] | Post | 2.65 [-3.11, 8.40] | 6.84 [0.92, 12.76] |
|  | FU | 8.24 [1.21, 15.26] | 12.03 [4.74, 19.32] |
| Hyperactivity EMM [95% CI] | Post | 1.37 [-4.68, 7.42] | 7.12 [1.35, 12.88] |
|  | FU | 5.64 [-0.60, 11.88] | 10.62 [3.92, 17.32] |
| Inattention EMM [95% CI] | Post | 2.37 [-3.72, 8.46] | 4.43 [-1.64, 10.50] |
|  | FU | 8.73 [0.55, 16.91] | 9.42 [1.87, 16.98] |

**Table S4:** Estimated marginal means (EMMs) and 95% confidence intervals (CIs) from ANCOVA models evaluating improvement in ADHD Total Symptoms, hyperactivity and inattention scores across ccPAS order groups at post-treatment (Post) and follow-up (FU), adjusted for baseline symptom severity.

**Figure S3: Influence of biomarker-based allocation to HF treatment on symptom scores: individual trajectories.** Plots depicting individual (thin lines) and mean (thick lines) trajectories of CAARS total ADHD symptom scores (A) and ADHD index (B) across the different HF allocation groups. Note that raw trajectories do not account for baseline symptom severity. Consequently, visual differences in symptom change may not fully reflect the baseline-adjusted effects estimated by the statistical models.

**Figure S4: Influence of biomarker-allocation to HF treatment on secondary outcome measures**. (A) Bar graphs of the ANCOVA model estimated means of improvement (±SEM) on the CAARS ADHD subscales for the HF allocation groups, controlling for baseline symptom levels. (B) Same for the subscales of BRIEF-A. End: end of treatment, FU: follow-up.

**Figure S5: Influence of ccPAS treatment on ADHD symptoms: individual trajectories.** Plots depicting individual (thin lines) and mean (thick lines) trajectories of CAARS total ADHD symptom scores (A), Hyperactivity-impulsivity scores (B) and inattention scores across ccPAS order groups.

**Figure S6: Influence of ccPAS on secondary outcome measures**. (A) Bar graphs of the ANCOVA model estimated means of improvement (±SEM) on the CAARS ADHD subscales for the ccPAS order groups (LR, RL), controlling for baseline symptom levels. (B) Same for the stopping times (SSRTs) of the Stop Signal task at the end of treatment (C) Same for the subscales of BRIEF-A. End: end of treatment, FU: follow-up. *p<0.05

References

Alyagon, U., Shahar, H., Hadar, A., Barnea-Ygael, N., Lazarovits, A., Shalev, H., Zangen, A., 2020. Alleviation of ADHD symptoms by non-invasive right prefrontal stimulation is correlated with EEG activity. NeuroImage Clin. 26, 102206. https://doi.org/10.1016/j.nicl.2020.102206

Berger, A., Alyagon, U., Hadaya, H., Atzaba-Poria, N., Auerbach, J.G., 2013. Response inhibition in preschoolers at familial risk for attention deficit hyperactivity disorder: a behavioral and electrophysiological stop-signal study. Child Dev. 84, 1616–1632. https://doi.org/10.1111/cdev.12072

Hadar, A., Hadas, I., Lazarovits, A., Alyagon, U., Eliraz, D., Zangen, A., 2017. Answering the missed call: Initial exploration of cognitive and electrophysiological changes associated with smartphone use and abuse. PLOS ONE 12, e0180094. https://doi.org/10.1371/journal.pone.0180094

Jung, T.-P., Makeig, S., Humphries, C., Lee, T.-W., McKEOWN, M.J., Iragui, V., Sejnowski, T.J., 2000. Removing electroencephalographic artifacts by blind source separation. Psychophysiology 37, 163–178. https://doi.org/10.1111/1469-8986.3720163

Rogasch, N.C., Thomson, R.H., Farzan, F., Fitzgibbon, B.M., Bailey, N.W., Hernandez-Pavon, J.C., Daskalakis, Z.J., Fitzgerald, P.B., 2014. Removing artefacts from TMS-EEG recordings using independent component analysis: importance for assessing prefrontal and motor cortex network properties. NeuroImage 101, 425–439. https://doi.org/10.1016/j.neuroimage.2014.07.037
